# The Short-Term Efficacy of Mulligan Traction Straight Leg Raise on Low Back Pain Associated With Hamstring Tightness in Young Adults

**DOI:** 10.7759/cureus.80215

**Published:** 2025-03-07

**Authors:** Aarya S Mane, Trupti Yadav

**Affiliations:** 1 Physical Medicine and Rehabilitation, Krishna College of Physiotherapy, Krishna Vishwa Vidyapeeth, Karad, IND

**Keywords:** hamstring tightness, low back pain, mulligan’s method, traction straight leg raise, young adults

## Abstract

Background and objective

Low back pain (LBP) is a very common problem faced by people globally; with a prevalence rate of almost 84%, it is nowadays commonly seen in the young adult population too. This population is also prone to a sedentary lifestyle, leading to hamstring tightness. As this population has very little time to spare in terms of long-term treatment, the Mulligan traction straight leg raise (TSLR) technique might be ideal for them as it is effective even in a short amount of time and may help treat LBP and hamstring tightness in a noninvasive manner and improve flexibility simultaneously.

Materials and methods

For LBP, an assessment of the pain rating scale was performed. For hamstring tightness, the active knee extension test (AKET), V-sit and reach test, and fingertip-to-floor test were employed with the help of a goniometer, plinth, inch tape, and chalk. Mulligan TSLR was performed for three consecutive days. After three days, an assessment was conducted to record the level of improvement.

Results

The investigations were performed in 100 young adults of which 60 (60%) were females and 40 (40%) were males. Normal BMI was observed in 67 (67%) participants while the remaining 33 (33%) participants were in the overweight category. LBP and hamstring tightness decreased after the application of the treatment, and the difference with the pre-treatment situation was statistically significant(p<0.0001). This signifies that the treatment is effective.

Conclusions

The present study demonstrated a significant reduction in LBP associated with hamstring tightness after the short-term application of Mulligan TSLR. Our findings show that this method alone can alleviate acute pain and tightness, leading to improvement in range of motion and muscle flexibility.

## Introduction

Low back pain (LBP) is one of the most significant health issues faced by today's generation, affecting a staggering amount of 568 million individuals, thereby restricting their activities of daily living and reducing their quality of life [1]. LBP refers to the pain experienced in the lumbar region of the spine which affects almost every individual at least once in their lifetime. According to the World Health Organization (WHO), the prevalence of LBP is alarmingly high, with estimates showing that up to 84% of the population is affected by it. This number is expected to continue to rise due to various contributing factors associated with modern lifestyles [2]. Several factors contribute to the development of LBP, such as obesity or increased weight, genetic predisposition (family history), stress, smoking, strenuous activity, and sedentary lifestyle or prolonged sitting (>5 hours). Due to LBP, some people experience limitations in activities of daily living. Moreover, the emotional toll of persistent pain can lead to feelings of depression and anxiety, further affecting the quality of life and work productivity [3,4].

The hamstring muscles, located in the posterior part of the thigh, play an important role in the function and movement of the lower body. The hamstrings consist of three main muscles: the semitendinosus, semimembranosus, and biceps femoris. These muscles are classified as two-joint muscles as they cross both the knee and hip joints, performing the vital functions of knee flexion and hip extension [5]. Shortening of the hamstring muscle leads to hamstring tightness. Hamstring tightness is quite common due to various reasons such as prolonged sitting or standing, and a sedentary lifestyle, which eventually leads to muscle imbalances and potential postural issues, making one prone to injury. Prolonged sitting can be seen in young adults due to academic and professional demands, which makes their age group more prone to hamstring tightness. This shortening of muscles eventually weakens the core, increases the risk of recurrent injury, and restricts the mobility of the pelvis, leading to alterations in lumbar pelvic rhythm [6,7].

The term young adults refers to the population in the age group of 18-25 years. This age group is in their third decade of life, making them more prone to LBP as many individuals within this group are college students or young professionals who spend the majority of their day sitting for long hours, whether in classrooms, at desks, or in front of computer screens [8]. Currently, this population is leading a lifestyle characterized by minimal physical activity, and sitting with a flexed knee for long hours a day, which causes shortening of the hamstring, leading the pelvis to tilt posteriorly, and lumbar lordosis resulting in LBP due to back muscle strain. In this crucial phase of life, young adults are often unaware of the long-term consequences of such poor posture and inactivity [5-9].

Fortunately, many of these issues can be prevented or mitigated through the adoption of an active and healthy lifestyle, as well as early intervention when pain or discomfort first arises. If the symptoms are chronic or severe, seeking appropriate treatment can significantly improve the overall quality of life. One such treatment option is Mulligan Traction Straight Leg Raise (TSLR): a technique invented by Brian Mulligan, which is a painless intervention with immense benefit for LBP and radiculopathy. There are various methods for the treatment of LBP, such as Mobilization With Movement (MWM) of extremities and Spinal Mobilization With Limb Movement (SMWLM) [10,11]. TSLR is an MWM technique that can be used as an option for improving hamstring flexibility, which may benefit people with chronic LBP.

This study aims to investigate whether the TSLR technique alone can effectively reduce LBP linked to hamstring tightness in a short period, while also promoting long-lasting effects that can enhance mobility and help reduce the risk of future pain episodes. By exploring the potential of this technique, the research seeks to offer evidence-based solutions to manage individuals suffering from the combined challenges of LBP and hamstring tightness, ultimately improving both physical function and overall well-being. By assessing the immediate changes in pain intensity and hamstring flexibility, this study seeks to provide insights into the potential clinical benefits of incorporating this technique into treatment plans for individuals suffering from this common, yet often under-recognized, musculoskeletal problem.

Given the high prevalence of LBP and hamstring tightness in young adults and the potential for substantial impairments in quality of life, understanding the effectiveness of manual therapy interventions like Mulligan TSLR could inform both clinical practice and patient care strategies. We believe our findings can help enhance treatment protocols for young adults, by providing a noninvasive and accessible option to improve both pain management and flexibility. They may also contribute to the broader body of evidence supporting the use of the Mulligan techniques as a viable treatment for musculoskeletal pain, particularly in younger, active populations.

## Materials and methods

The current study of the short-term effectiveness of Mulligan TSLR on LBP associated with hamstring tightness in young adults was carried out at Krishna College of Physiotherapy, Krishna Vishwa Vidyapeeth (KVV), Karad. Certification was obtained from the protocol committee, along with approval from authorities and the ethical committee. Participants were selected according to inclusion and exclusion criteria.

The sample size for this study was calculated with the help of the statistician who used the formula n = 4pq/L^2 ^to calculate the sample size. Here p is the prevalence of hamstring tightness and LBP, i.e., 61 % according to the study by Batool et al. [12]; q = 100-p; and L is the allowable error, i.e., 10%. After the calculation, the value achieved was 95.16. We rounded it off and the sample size was set to 100.

Of the total 150 students, 100 fulfilled the inclusion criteria of having LBP associated with hamstring tightness, and the rest were excluded. Informed consent was taken from participants, and the demographic data were documented on the first day. Outcome measures were taken before treatment on the first day and after treatment on the third day. A master chart was prepared using all the relevant data. Statistical analysis was performed with the help of the statistician, after which the mean of both groups pre and post was calculated and graphs were prepared from the retrieved data. 

Inclusion criteria

Young adults aged 18-25 years of both genders with LBP and hamstring tightness.

Exclusion criteria

People with recent falls or fractures, surgeries, or lumbar pathologies (eg. degenerative disc diseases, ankylosing spondylitis).

Assessment tools

The numerical pain rating scale (NPRS) [13] was used to assess LBP; for hamstring tightness, special tests like the active knee extension test (AKET) [14], The V-sit and reach test [15], and fingertip-to-floor test [16] were used for assessment. LBP and hamstring tightness were measured pre- and post-treatment. Mulligan TSLR [17] was given to the selected participants for three consecutive days. Post-treatment assessment was done, data were collected, and statistical analysis was done.

Treatment protocol

The subjects were given Mulligan TSLR [17] for three consecutive days without combining it with any other treatment. This protocol was administered by one of the authors (a physiotherapist) in a clinic setting under the guidance of a trained physiotherapist: three repetitions on the first treatment occasion, six repetitions on the second day, and nine repetitions on the third day.

Therapist position: standing beside the patient’s treating side in a lunge stance and facing the patient’s head.

Patient position: supine on a low plinth or floor.

**Figure 1:**
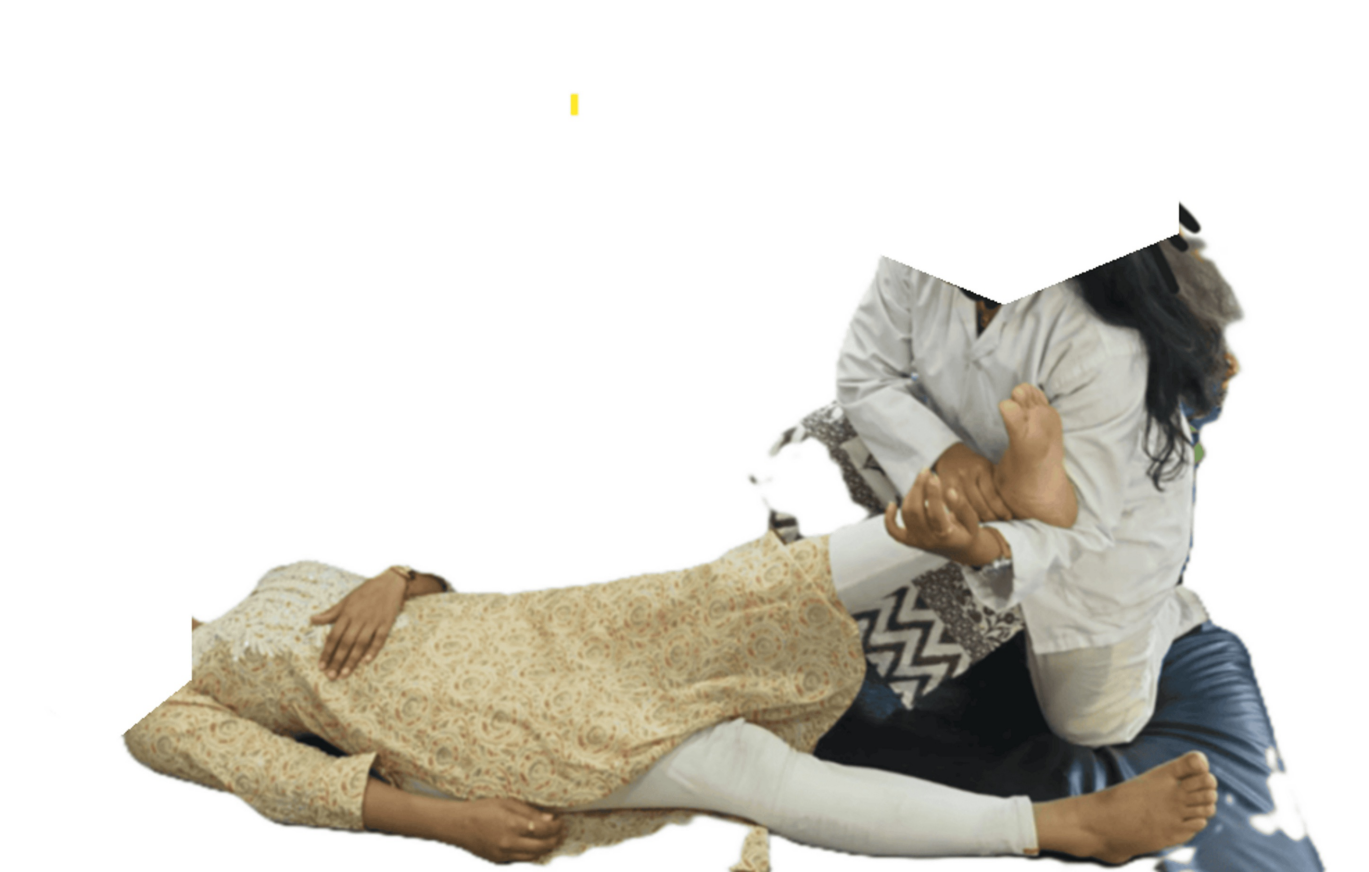
Mulligan TSLR - image 1 The therapist applies the Mulligan TSLR TSLR: traction straight leg raise

**Figure 2:**
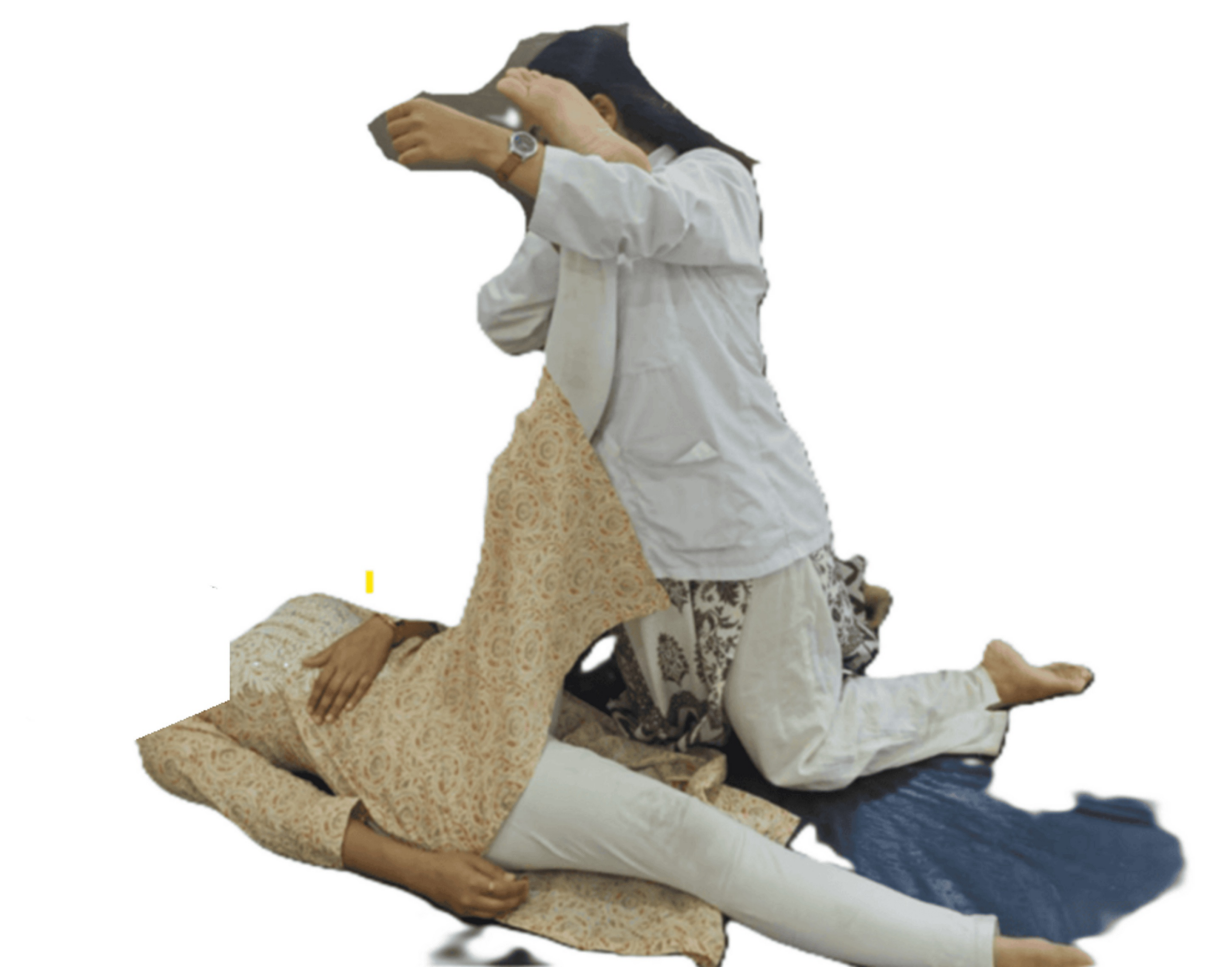
Mulligan TSLR - image 2 The therapist has given traction and is maintaining it for 10 seconds TSLR: traction straight leg raise

Procedure: the patient’s leg was grasped proximal to the ankle, in the crook of the therapist’s elbow with the other hand grasping the anterior shin. A longitudinal glide was applied along the line of the femur, by extending the knees and leaning backward. While maintaining the glide, the patient's leg was moved passively into the pain-free range of SLR. The traction was applied for 10 seconds (Figures 1, 2).

## Results

This study included 100 participants, of which 40 (40%) were male and 60 (60%) were female. The young adults who had LBP along with hamstring tightness were selected. These individuals were assessed, treated only with Mulligan TSLR, and then were reassessed to examine the level of improvement.

Table 1 depicts the demographic characteristics of the study cohort. Fifty participants (52%) participants were in their early 20s, i.e., in the age group of 21-23 years. The least number of people were in the age group of 24-25 years: 19 (19%). The late teens or the age group of 18-20 years comprised 32 (32%) participants. The majority of the participants (67, 67%) had a normal BMI. While none of the participants were underweight or obese, a few (33, 33%) were overweight. Figure 1 illustrates the gender distribution in the study cohort.

**Table 1:** Demographic data of the participants BMI: body mass index

Parameters	Frequency (percentage)
Age group, years	
18-20	32 (32%)
21-23	52 (52%)
24-25	19 (19%)
Gender	-
Male	40 (40%)
Female	60 (60%)
BMI, kg/m^2^	-
18-24.9	67 (67%)
25-30.9	33 (33%)

**Figure 3:**
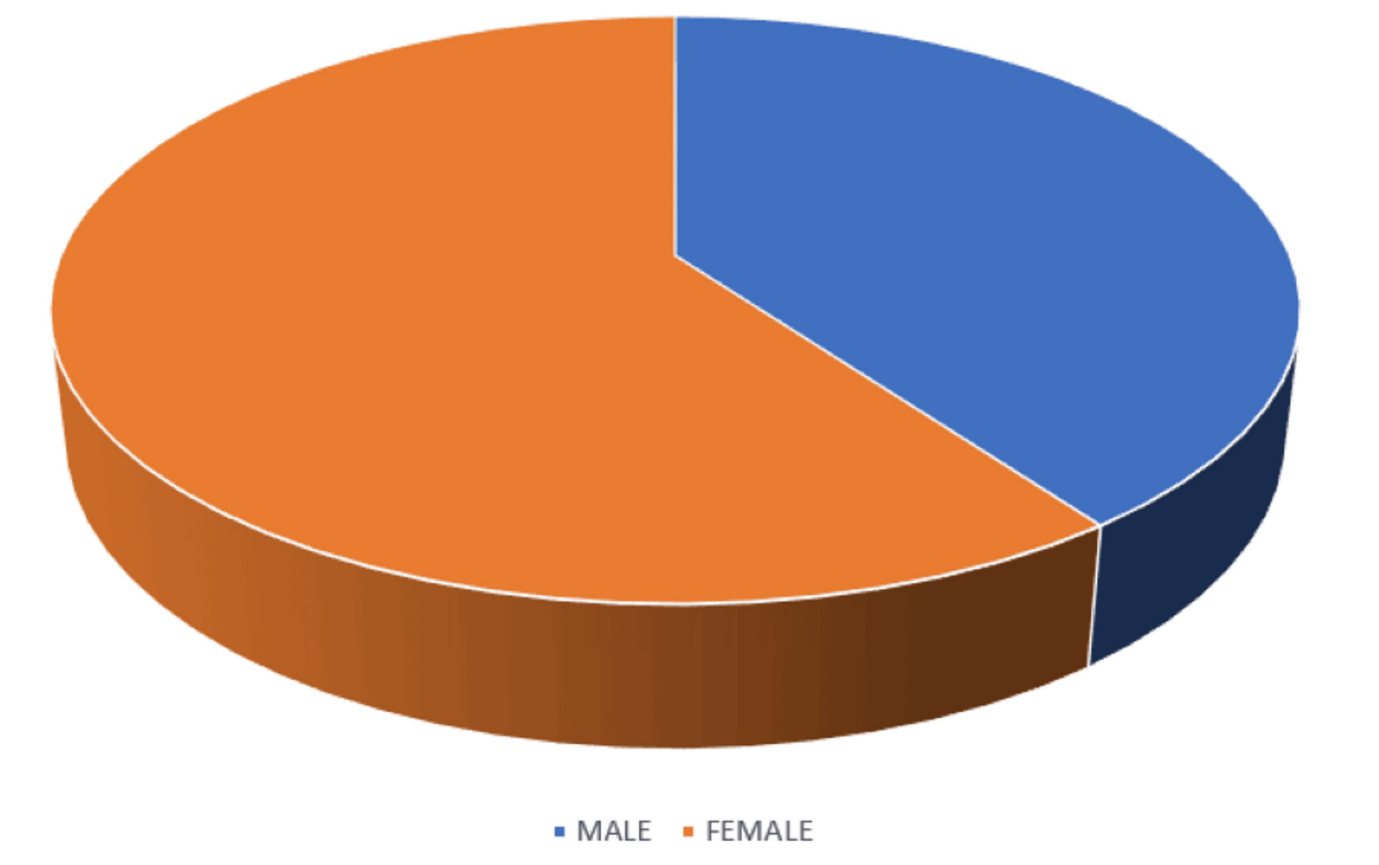
Gender distribution in this study

Table 2 details the results based on various assessment tools. Regarding the pain rating scale [13], there was almost no difference in values at rest vs. on activity, which concludes that the pre-treatment values are homogeneous. The pain is drastically reduced post-treatment, especially during activity. As for AKET [14], it is seen that the right side is slightly more affected than the left pre-treatment, but both ranges improve post-treatment. While the V-sit and reach test [15] and the fingertip-to-floor test [16] also show almost the same tightness pre-treatment, the fingertip-to-floor test shows slightly more improved results than the other test. There was a significant improvement in LBP and hamstring tightness after the intervention, which illustrated the short-term effectiveness of Mulligan TSLR [17]. Figure 2 provides a comparative analysis of the outcome measures pre- and post-treatment.

**Table 2:** Analysis of outcome measures before and after the treatment AKET: active knee extension test; NPRS: numerical pain rating scale; SD: standard deviation

Outcome measures	Pre-treatment		Post-treatment		P-value	T-value
-	Mean	SD	Mean	SD	-	-	
NPRS (at rest)	4.01	1.419	0.65	0.8211	<0.0001 (significant)	22.692	
NPRS (on activity)	4.23	1.406	0.52	0.6273	<0.0001 (significant)	24.097	
AKET (left)	29.5	4.439	17.46	2.091	<0.0001 (significant)	24.536	
AKET (right)	30.69	5.136	17.54	2.329	<0.0001 (significant)	23.319	
V-sit and reach test	-3.968	2.189	-0.795	1.019	<0.0001 (significant)	13.140	
Fingertip-to-floor test	3.773	1.843	0.669	0.8598	<0.0001 (significant)	15.261	

**Figure 4:**
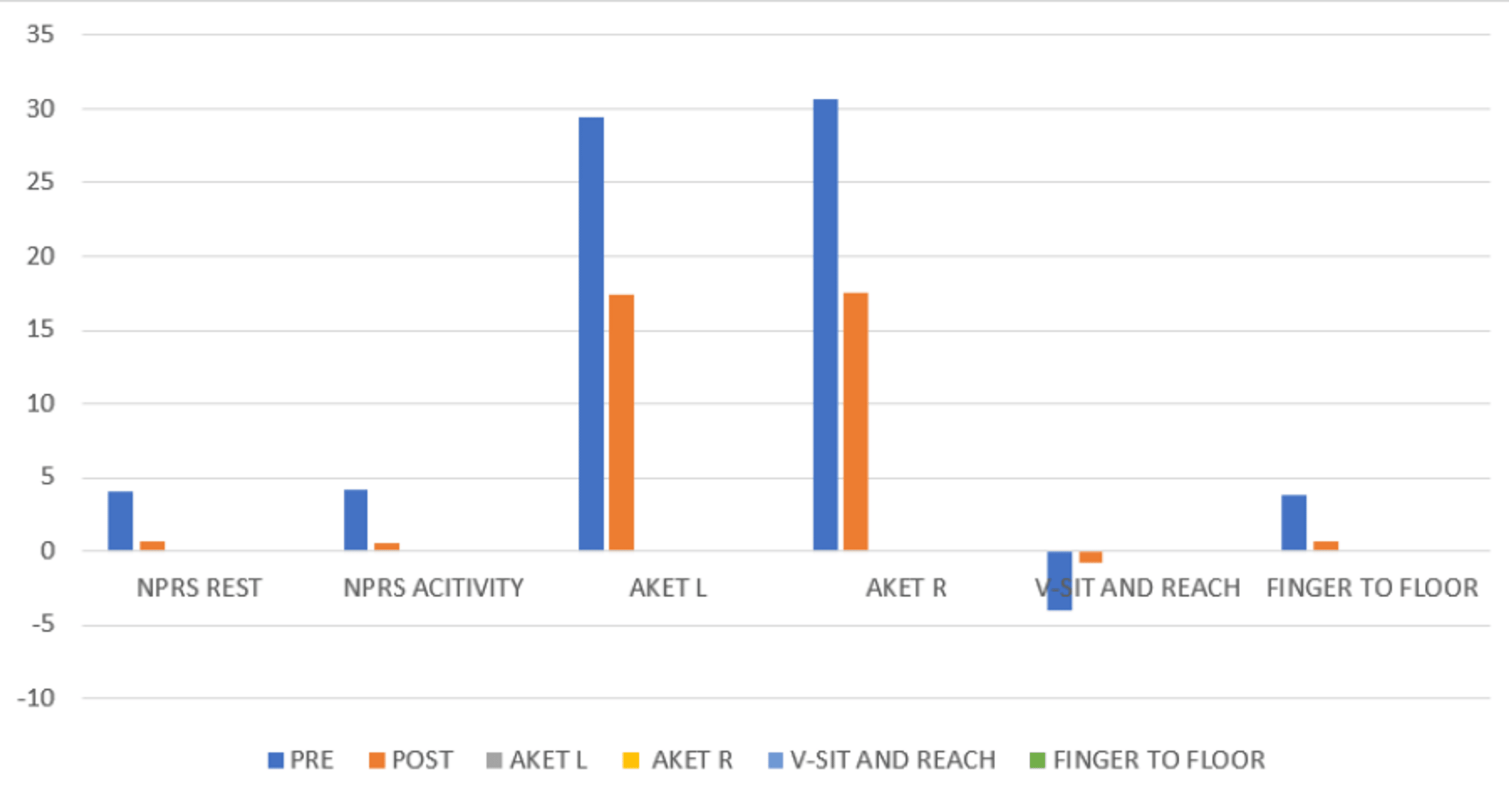
Comparison of outcome measures pre- and post-treatment The graph indicates the reduction in low back pain associated with hamstring tightness, i.e., improvement in the condition AKET: active knee extension test; NPRS: numerical pain rating scale

## Discussion

The current study aimed to verify whether there was any short-term effectiveness of applying Mulligan TSLR on LBP associated with hamstring tightness in young adults. In our study, the majority (60, 60%) were females and the rest 40 (40%) were males. This is because the study center had a majority female population. Batool et al. in their study [12] concluded that females are more likely to be disabled due to LBP and hamstring tightness than males due to the anatomical differences and lower mineral density, which can also be one of the major contributing factors. This factor needs to be further evaluated as it was not considered in the present study. Young adults were picked for this study since they are the most prevalent demographic in terms of LBP [3]. More than half of the population (52, 52%) participants were in the age group 21-23 years. The age group of late teens (18-20) consisted of 32 (32%) participants and that of mid-20s (24-25 years) accounted for only 19 (19%).

Young adults are a group of people who commonly tend to avoid physical activity, resulting in a sedentary lifestyle, which eventually leads to tight hamstrings and lower back pain. At this age, most of the population is career-focused, and they consequently ignore physical activity, leading to prolonged sitting. Acute LBP can progress to a chronic form, which eventually affects the productivity of these young adults. Similar findings were reported by Kamalakannan et al. in their study [7]. The proportion of subjects experiencing LBP who were sedentary was 74%, while those who did physical exercise were only 26%, as reported in the study by Furtado et al. [4].

To associate LBP with hamstring tightness, screening was done by taking a thorough history. People with LBP due to pathology were eliminatedThe study by Nelakurthy et al. stated that a shortening of hamstring muscle, which can be due to various reasons, eventually leads to posterior pelvic tilt; this tilt leads to reduced lordotic curve, alters spinal loading, and results in the alteration in the lumbar pelvic rhythm, which generates strain on the lumbar segment, causing muscle impairment giving rise to LBP [18]. There is an alteration in the biomechanics, and, to compensate for this, wrong alterations are again made, which lead to tightness, discomfort, and a complex feedback loop, thereby worsening the situation [7]. Batool et al. described the link between chronic LBP and stiffness in the back of the thigh in their study [12]. They found that hamstring tightness leads to chronic LBP and functional disability. Treating hamstring tightness may reduce LBP. The same correlation was reported by Gou et al. in their study [1]. They stated that addressing the condition in one area can alleviate pain and enhance functionality in the untreated area. In their study, it was observed that individuals with LBP had weak core muscles associated with hamstring tightness and coordination impairment; however, improving hamstring flexibility and core strength will help enhance spine stability.

The association between LBP and hamstring tightness can be due to multiple reasons such as muscle imbalances, facet joint irritation, and nerve root compression. This compression can be due to repetitive motions or disc herniation. In our study, LBP and hamstring tightness were assessed before treatment and after three days of treatment. Based on the data collected, the difference in outcomes was significant (p<0.0001), and hence it was concluded that the treatment was effective. When Mulligan TSLR is applied, there is activation of mechanoreceptors, which inhibits pain and increases hip flexion range, leading to a reduction of mechanical stress on the painful lumbar structures during the straight leg raise. This method helps reduce tightness, improve movement patterns in the lower back, and help restore neutral spinal alignment. This was explained by Hall et al. in their study [11]. This study showed that three days of TSLR alone is sufficient for treating LBP associated with hamstring tightness. In this study, there was a significant reduction in LBP and hamstring tightness.

While the immediate effects of Mulligan TSLR on pain and flexibility were significant, it is important to note that the long-term impact of this intervention was not assessed in this study. The results of this study provide valuable insights into the short-term benefits of Mulligan TSLR for young adults with LBP associated with hamstring tightness. These findings suggest that Mulligan TSLR can be a useful tool for clinicians, seeking to provide quick relief for patients experiencing this common musculoskeletal issue.

This study has a few limitations, primarily its small sample size, which may limit the generalizability of the findings. While the results showed positive outcomes, larger studies with more diverse populations are needed to confirm the broader applicability of these findings. Additionally, this study focused on the short-term effects of Mulligan TSLR, and long-term follow-up was not conducted to determine the sustainability of the intervention's benefits.

## Conclusions

Based on our findings among young adults, a sedentary lifestyle is the main cause of hamstring tightness, which eventually leads to LBP. The patients in the age group of 18-25 do not generally maintain a proper posture or practice posture-correction techniques. Our findings showed that the short-term application of Mulligan TSLR can help achieve a reduction in LBP and hamstring tightness. Administering this technique over a three-day protocol period helped in reducing the LBP of the patients significantly. This method can single-handedly alleviate acute pain and tightness, leading to improvement in range of motion and muscle flexibility, and should be considered as a treatment option for LBP and hamstring tightness in young adults.
